# A Novel Implementation of Physiotherapy in a Known Case of Malunited Supracondylar Fracture of the Femur With Osteomyelitis Managed With Ilizarov Fixator

**DOI:** 10.7759/cureus.30853

**Published:** 2022-10-29

**Authors:** Purva S Shahade, Purva H Mundada, Ruchika J Zade, Pratik Phansopkar

**Affiliations:** 1 Physiotherapy, Ravi Nair Physiotherapy College, Datta Meghe Institute of Medical Sciences, Wardha, IND; 2 Neurophysiotherapy, Ravi Nair Physiotherapy College, Datta Meghe Institute of Medical Sciences, Wardha, IND; 3 Musculoskeletal Physiotherapy, Ravi Nair Physiotherapy College, Datta Meghe Institute of Medical Sciences, Wardha, IND

**Keywords:** proximal tibia-fibula fracture, foot drop, ilizarov ring fixator, rehabilitation, osteomyelitis, supracondylar femur fracture

## Abstract

The purpose of this case study was to elucidate the significance of physiotherapy management in rehabilitating an individual with osteomyelitis. The patient was a 25-year-old male with complaints of purulent discharge from wounds above the knee. The physiotherapy intervention prescribed and noted here focuses on enhancing functional goals during the postoperative phase. These therapeutic interventions revolve around functional exercises, which will ultimately help and assist the patient gain independence and enhance the patient’s cardiovascular capacity. This case report focuses on the mandatory novel implementation of physical therapy interventions in an operated case of malunited supracondylar fracture of the femur of 10-month duration with osteomyelitis and proximal tibia-fibula fracture with right-sided foot drop managed with an Ilizarov external ring fixator.

## Introduction

In 1834, Nelaton was the first to coin the term “osteomyelitis” [1]. Osteomyelitis is an infection caused by bacteria (streptococci and staphylococci), viruses, or fungi that leads to the progression of inflammatory destruction of bone along with the bone marrow [2]. It can be classified as an infection secondary to contagious focus or following hematogenous spread or a consequence of vascular insufficiency (e.g., diabetic foot), and it usually occurs as a secondary complication to contagious infections caused by surgery or trauma [3]. The bone infection leads to the formation of exudate, which eventually leads to the formation of soft tissue abscess. Due to all this, there is impaired blood flow, resulting in the production of a dead bone piece that later gets separated from the healthy part of the bone [4].

Osteomyelitis is visible mainly in those kinds of open fractures that are contaminated grossly and in those sorts of fractures that have undergone the procedure of internal fixation. The percentage of risk of osteomyelitis, which is trauma-induced in long open bone fractures, ranges somewhere between 3% and 50% based on its severity level, with the recurrence rates approximately as high as 20%-30% [5]. Chronic osteomyelitis usually lasts longer, and the patient is susceptible to repeated attacks [6]. The incidence of osteomyelitis has been greatly reduced because of the introduction of various antisepsis [7].

This case study is of a 25-year-old male who was diagnosed with a 10-month-old infected malunited fracture of the supracondylar femur with osteomyelitis and a proximal tibia-fibula fracture with foot drop on the right side managed with an Ilizarov external ring fixator. Our case study focuses on the mandatory novel implementation of physical therapy assessment and intervention strategies for the rehabilitation of this case.

## Case presentation

Patient information

**Figure 1:**
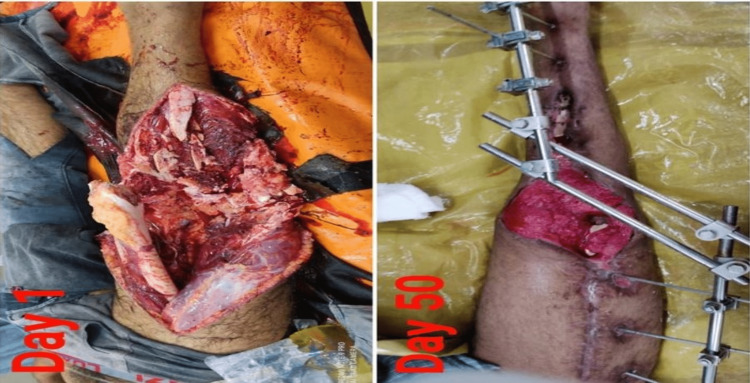
Picture at the time of injury and after surgery (application of an external fixator)

A 25-year-old male patient, a resident of Nagpur district and an owner of a small business vehicle, had given an alleged history of a road traffic accident (RTA) as he had been hit by a four-wheeler while he was riding a two-wheeler on November 9, 2020, during which he sustained an injury on the right lower limb. Immediately after the injury, the patient was unable to bear weight over the right lower limb. He was immediately taken to a nearby hospital in Nagpur where an external fixator was applied on November 10, 2020. He was later referred to another hospital for plastic surgery. The patient then presented to Acharya Vinoba Bhave Rural Hospital (AVBRH) orthopedics outpatient department (OPD) with complaints of a wound above the knee of the right lower limb for five months, and he also had purulent discharge from the wound for the past seven days. There was no history of loss of consciousness/headache and no history of vomiting and ear, nose, or throat (ENT) bleeding. He was operated in AVBRH for the Ilizarov ring fixator. He underwent three operations: skin grafting for the wound over the right thigh, Ilizarov ring realignment on the right side, and open reduction and internal fixation (ORIF) with plating for the right distal femur and proximal tibia. The picture at the time of injury and after surgery (application of an external fixator) is shown in Figure 1.

Clinical findings

**Figure 2:**
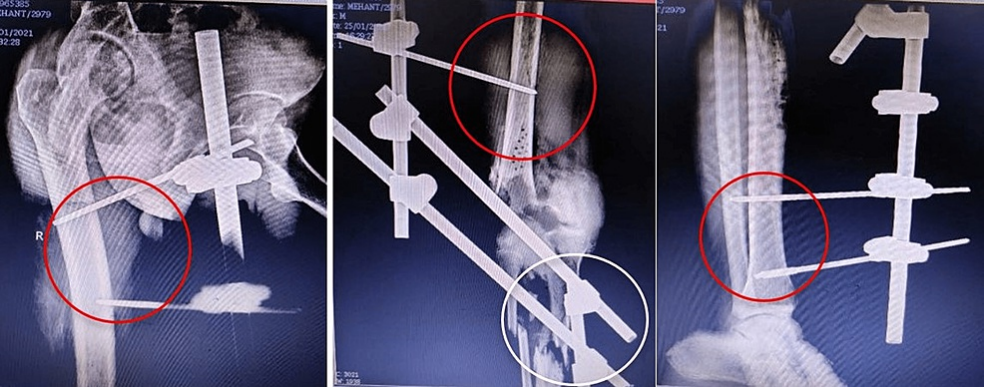
Postoperative X-ray where an external fixator was applied

After obtaining consent, the patient was taken for examination. He was examined in a long sitting position with both shoulders at a similar level. His right lower limb was covered in a plaster slab with his knee slightly flexed and supported over a pillow and an externally rotated hip. No movement was possible in the right knee joint. He was advised not to bear weight on the operated right leg. A postoperative X-ray is given in Figure 2.

On observation, the body build was ectomorph, and the posture was normal. Pretreatment on the visual analog scale (VAS), the patient rated the pain as 9/10 at rest and 10/10 on slight movement. The range of motion (ROM) was restricted due to pain (Table 1). Manual muscle testing (MMT) pre-intervention is given in Table 2.

**Table 1:** Range of motion assessment pre-intervention

Joint	Right active	Right passive	Left active	Left passive
Hip				
Flexion	0-3˚	0-7˚	0-110˚	0-110˚
Extension	0-1˚	0-3˚	0-19˚	0-24˚
Knee				
Flexion	0-5˚	0-10˚	0-110˚	0-119˚
Extension	25-0˚	30-0˚	119-95˚	120-94˚
Ankle				
Plantar flexion	0˚	0˚	0-33˚	0-36˚
Dorsiflexion	0˚	0˚	0-8˚	0-14˚

**Table 2:** Manual muscle testing pre-intervention

Manual muscle testing		
Muscles	Right	Left
Hip		
Flexors	1/5	4/5
Extensors	1/5	4/5
Knee		
Flexors	1/5	4/5
Extensors	1/5	4/5
Ankle		
Plantar flexors	1/5	4/5
Dorsiflexors	1/5	4/5

Medications

Medications with dosage are given in Table 3.

**Table 3:** Medications with dosage

Medication	Dosage
Tablet linezolid	300 mg 12 hourly daily
Tablet pantoprazole	40 mg 24 hourly daily
Tablet paracetamol	650 mg 12 hourly daily
Tablet calcium	500 mg 12 hourly daily
Tablet vitamin C	500 mg 12 hourly daily

Physiotherapy management

Physiotherapy treatment was administered to the patient for four weeks. The rehabilitation goals were independent non-weight-bearing walking with a walker, minimal assistance for activities of daily living (ADLs), maintaining the muscle integrity of the right lower limb, and respiratory care. Detailed physiotherapy management along with a home exercise program is given in Table 4.

**Table 4:** Phase-wise physiotherapy rehabilitation along with home exercise program mins: minutes, reps: repetitions, sec(s): second(s), ROM: range of motion, ADLs: activities of daily living

Intervention	Rationale	Intensity	Instructions	Phase 1 (week 0-3)	Phase 2 (week 3-6)	Phase 3 (week 6-9)	Phase 4 (week 9-12)	Home exercise program
Elevation with the help of a pillow to the right lower limb	To enhance the circulation of blood and the healing process	The angle at which the limb is elevated and the duration are decided as per the tolerance level of the patient	The patient was asked to relax and inform the therapist if discomfort occurred; the pillow under the limb is usually at 30° of elevation	10° of elevation/two hourly and then relaxation of 15 mins	20° of elevation/two hourly and then relaxation of 15 mins	25° of elevation/two hourly and then relaxation of 15 mins	30° of elevation/two hourly and then relaxation of 15 mins	30° of elevation/two hourly and then relaxation of 15 mins
Ankle toe movements	For enhancing the proper circulation of blood and the healing process	As per the tolerance level of the patient and the need for an intervention	The patient was asked to dorsiflex and plantar flex the foot as if pedaling a bicycle	Five reps - one set/thrice a day - gravity-eliminated position - supine	10 reps - one set/thrice a day - gravity-eliminated position - supine	10 reps - one set/thrice a day – gravity-assisted and gravity-resisted position - bedside sitting	10 reps - one set/thrice a day – gravity-assisted and gravity-resisted position - with minimal resistance by therapist’s hand - bedside sitting	10 reps - one set/thrice a day – gravity-assisted and gravity-resisted position - with moderate resistance by therapist’s hand or a theraband - bedside sitting
Isometric contraction for right hamstrings	For strengthening the hamstring	As per the tolerance of the patient	The patient was asked to apply half of his muscle power, and the therapist applied a similar amount of muscle force but in the opposite direction so that there was not any movement but strengthening occurred	Five reps with 5-sec hold - thrice a day	Seven reps with 5-sec hold - thrice a day	Seven reps with 10-sec hold - thrice a day	10 reps with 10-sec hold - thrice a day	15 reps with 10-sec hold - thrice a day
Isometric contraction for right quadriceps	For strengthening the quadriceps	As per the tolerance of the patient	The patient was asked to apply half of his muscle power, and the therapist applied a similar amount of muscle force but in the opposite direction so that there was not any movement but strengthening occurred	Five reps with 5-sec hold - thrice a day	Seven reps with 5-sec hold - thrice a day	Seven reps with 10-sec hold - thrice a day	10 reps with 10-sec hold - thrice a day	15 reps with 10-sec hold - thrice a day
Isometric contraction for right gluteus muscles	For strengthening the right gluteus muscles	As per the tolerance of the patient	The patient was asked to apply half of his muscle power, and the therapist applied a similar amount of muscle force but in the opposite direction so that there was not any movement but strengthening occurred	Five reps with 5-sec hold - thrice a day	Seven reps with 5-sec hold - thrice a day	Seven reps with 10-sec hold - thrice a day	10 reps with 10-sec hold - thrice a day	15 reps with 10-sec hold - thrice a day
Active resisted exercises for upper limbs	For preventing contractures and for maintaining the ROM as well as the strength	As per the tolerance of the patient	The patient was asked to do it with the help of weight cuffs or theraband and as per the tolerance level	Five reps - per set - thrice a day - against gravity	Seven reps - per set - thrice a day - against gravity along with half kg weight cuff	Seven reps - per set - thrice a day - against gravity along with half kg weight cuff	10 reps - per set - thrice a day - against gravity along with one kg weight cuff	10 reps - per set - thrice a day - against gravity along with one kg weight cuff
Deep breathing exercises	To relax the patient	As per the comfort of the patient	The patient was instructed to relax in a comfortable position, and then, the deep breathing exercises were taught to the patient by the therapist	Five reps - per set - thrice a day, with 3-6-9 technique (3 secs - inspiration, 6 secs - hold the breath, 9 sec - expiration)	10 reps - per set - thrice a day, with 3-6-9 technique	10 reps - per set - thrice a day, with 3-6-9 technique	10 reps - per set - thrice a day, with 3-6-9 technique	10 reps - per set - thrice a day, with 3-6-9 technique
Sit-to-stand activities	To make the patient independent for his ADLs	As per the pain tolerance of the patient	The patient was instructed on how to progress from sitting to standing along with a mirror biofeedback	The patient was undergoing strengthening exercises, which are prerequisites for sit-to-stand activities	Five reps - one set - twice a day	Five reps - one set - thrice a day	10 reps - one set - thrice a day	15 reps - one set - thrice a day
Ambulation	To make the patient independent for his ADLs	As per the pain tolerance of the patient	The patient was instructed and taught how to use assistive devices - a walker along with a mirror biofeedback	The patient was undergoing strengthening exercises, which are prerequisites for ambulation	One round of 50 meters - with a walker - thrice a day	Two rounds of 70 meters - with a walker - thrice a day	Two rounds of 100 meters - with a walker - thrice a day	Three rounds of 100 meters - with a walker - thrice a day

Outcome

Post-rehabilitation VAS score was 3/10 at rest and 4/10 for slight movement. The patient could initiate active assisted movements of the right lower limb, and he could walk with the help of a walker with a strategy of non-weight-bearing. Also, the ROM of all joints of the lower limb has increased (Table 5 and Table 6).

**Table 5:** Range of motion assessment post-intervention

Joint	Right active	Right passive	Left active	Left passive
Hip				
Flexion	0-79˚	0-100˚	0-110˚	0-110˚
Extension	0-10˚	0-15˚	0-19˚	0-24˚
Knee				
Flexion	0-67˚	0-74˚	0-110˚	0-119˚
Extension	59-30˚	80-60˚	119-95˚	120-94˚
Ankle				
Plantar flexion	0˚-22	0˚-24	0-33˚	0-36˚
Dorsiflexion	0˚-3	0˚5	0-8˚	0-14˚

**Table 6:** Manual muscle testing post-intervention

Manual muscle testing		
Muscles	Right	Left
Hip		
Flexors	3/5	4/5
Extensors	2/5	4/5
Knee		
Flexors	3/5	4/5
Extensors	2/5	4/5
Ankle		
Plantar flexors	3/5	4/5
Dorsiflexors	3/5	4/5

## Discussion

Phansopkar et al. have studied a case and presented a case report of a nine-year-old male, a student in the fourth standard who was suffering from osteomyelitis. Their study concluded that a definitive surgical approach along with early physiotherapy rehabilitation was beneficial. An enhancement in functional goals was noted, which eventually led to the successful recovery of the patient [8].

Pande has presented a case report on chronic osteomyelitis, and he concluded that the management of chronic osteomyelitis is a difficult task, possibly because of the surgical procedures and antimicrobial therapies. Several methods for surgical reconstruction and the delivery of appropriate antibiotics are available. Staging along with the identification of the causative organisms is important for successful treatment [9].

A case study presented by Sipahioglu et al. in 2014 was about a five-year-old male suffering from bilateral acute tibial osteomyelitis without any underlying disease. This study concluded that early diagnosis along with proper antibiotic therapy and mandatory surgical implementation was important and beneficial [10].

A case study of a 25-year-old male who was diagnosed with a 10-month-old infected malunited fracture of the supracondylar femur with osteomyelitis with proximal tibia-fibula fracture with right-sided foot drop managed with Ilizarov external ring fixator is discussed here. This case study consists of the assessment and intervention strategies that were implemented. The assessment consists of body functions and structural impairments evaluated through a proper assessment of the respiratory system, cardiovascular system, ROM, and MMT. The therapeutic interventions include medications and physiotherapy management. Physiotherapy management includes respiratory care along with musculoskeletal management.

## Conclusions

This is a highly complicated case of osteomyelitis. A 25-year-old male has suffered a road traffic accident. He has been diagnosed with a 10-month-old infected malunited fracture of the supracondylar femur with osteomyelitis with proximal tibia-fibula fracture with right-sided foot drop managed with Ilizarov external ring fixator. Although it is a depressing condition for the patient as well as the family members, it is a relief that by designing a novel and anatomically proper physiotherapy management, the quality of life can be improved and independence level can be enhanced. A comprehensive physiotherapy rehabilitation protocol was designed taking into consideration all the conditions our patient had, and the patient was motivated throughout the rehabilitation sessions to stick to the given protocol and perform all the exercises consistently and appropriately, which resulted in improvements in movement ability, muscle strength, and balance, providing a safe return to independence in daily activities.
